# A case of hepatic segmental atrophy mimicking spontaneously necrotized hepatocellular carcinoma

**DOI:** 10.1016/j.radcr.2025.09.072

**Published:** 2025-10-30

**Authors:** Hitomi Kamiya, Eriko Yoshizawa, Akira Yamada, Mai Iwaya, Koji Kubota, Takefumi Kimura, Hidenori Ojima, Yasunari Fujinaga

**Affiliations:** a Department of Radiology, Shinshu University School of Medicine, 3-1-1 Asahi, Matsumoto City, Nagano 390-8621, Japan; b Medical Data Science Course, Shinshu University School of Medicine, 3-1-1 Asahi, Matsumoto City, Nagano 390-8621, Japan; c Department of Laboratory Medicine, Shinshu University Hospital, 3-1-1 Asahi, Matsumoto City, Nagano 390-8621, Japan; d Department of Surgery, Division of Gastroenterological, Hepato-Biliary-Pancreatic, Transplantation and Pediatric Surgery, Shinshu University School of Medicine, 3-1-1 Asahi, Matsumoto City, Nagano 390-8621, Japan; e Department of Medicine, Division of Gastroenterology, Shinshu University School of Medicine, 3-1-1 Asahi, Matsumoto City, Nagano 390-8621, Japan; f Division of Molecular Pathology, Department of Diagnostic Pathology, Tochigi Cancer Center 4-9-13 Yohnan, Utsunomiya, Tochigi 320-0834, Japan

**Keywords:** Segmental atrophy, Liver pseudotumor, Hepatocellular carcinoma, Spontaneous necrosis

## Abstract

We report a rare case of hepatic segmental atrophy that was initially suspected to be hepatocellular carcinoma (HCC). An 80-year-old man presented with a new 3 cm hepatic lesion that met LI-RADS-5 criteria. However, follow-up CT performed 1 month later showed loss of arterial hyperenhancement, progressive delayed peripheral enhancement, and a reduction in lesion size. MRI subsequently demonstrated high signal intensity on T2-weighted and diffusion-weighted images, without true diffusion restriction. Based on these imaging features, spontaneous necrosis of scirrhous-type HCC was suspected, and surgical resection was undertaken. Histopathological examination revealed hepatic segmental atrophy with no evidence of malignancy. Segmental atrophy can closely mimic HCC on imaging, potentially leading to misdiagnosis and unnecessary surgery. Careful evaluation of atypical features—such as the absence of true washout, lack of restricted diffusion, disproportionate capsule-like structures, and progressive morphological changes—may assist in distinguishing segmental atrophy from malignancy.

## Graphical abstract

**Figure:**
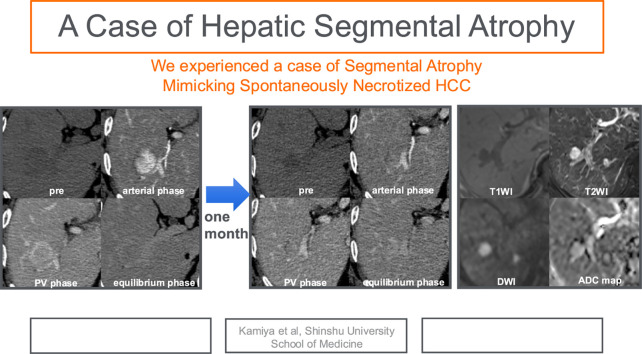

## Introduction

The liver can develop a range of benign and malignant lesions, and distinguishing between the 2 on imaging can be challenging, as their features often overlap. The most common type of primary liver cancer is hepatocellular carcinoma (HCC) [1]. To aid in the standardized assessment of liver lesions, the American College of Radiology has developed the liver imaging reporting and data system (LI-RADS) [2], which provides a structured algorithm for interpreting and reporting liver imaging findings. When specific imaging criteria are met, a lesion can be classified as LR-5, indicating it is “definitely HCC.” However, HCC can occasionally undergo spontaneous necrosis, leading to atypical imaging appearances [3]. In this case report, we present a patient whose initial imaging findings met LR-5 criteria, but whose lesion demonstrated significant changes over 1 month. Based on a presumptive diagnosis of HCC with spontaneous necrosis, surgical resection was performed. Histopathological examination, however, revealed a non-neoplastic lesion—hepatic segmental atrophy. We discuss key considerations for accurate preoperative imaging diagnosis through retrospective analysis of the major and ancillary LI-RADS features, and explore potential explanations for the atypical radiological evolution in light of pathologic–radiologic correlations.

## Case presentation

An 80-year-old man was referred to our institution after an incidental hepatic lesion was detected during an abdominal ultrasound performed as part of an annual health checkup. The patient had no symptoms. His medical history included primary biliary cirrhosis (PBC), which had been diagnosed several years prior, though he had discontinued regular medical follow-up and treatment. He also had hypertension under control with oral medication and a history of diabetes managed with diet therapy. His only surgical history was an appendectomy approximately 30 years earlier. There was no significant family history of liver disease or malignancy. Laboratory tests revealed that most liver function parameters were within the normal range: AST 29 U/L, ALT 37 U/L, serum albumin 4.1 g/dL, total bilirubin 1.45 mg/dL, and INR 1.03. However, γ-glutamyl transpeptidase (γ-GTP) was elevated at 266 U/L, and anti-mitochondrial M2 antibody was markedly elevated at 153.3 U/mL, consistent with underlying primary biliary cholangitis. Hyaluronic acid was also elevated at 91.5 ng/mL, suggesting the presence of liver fibrosis. Hemoglobin A1c was elevated at 7.9%, indicating poorly controlled diabetes mellitus. Hepatitis B and C markers were negative, and tumor markers including AFP, PIVKA-II, CEA, and CA19-9 were all within normal limits. Based on these findings, contrast-enhanced CT and magnetic resonance imaging (MRI) were performed to further evaluate the hepatic lesion and assess its nature.

### Imaging findings

**Fig. 1:**
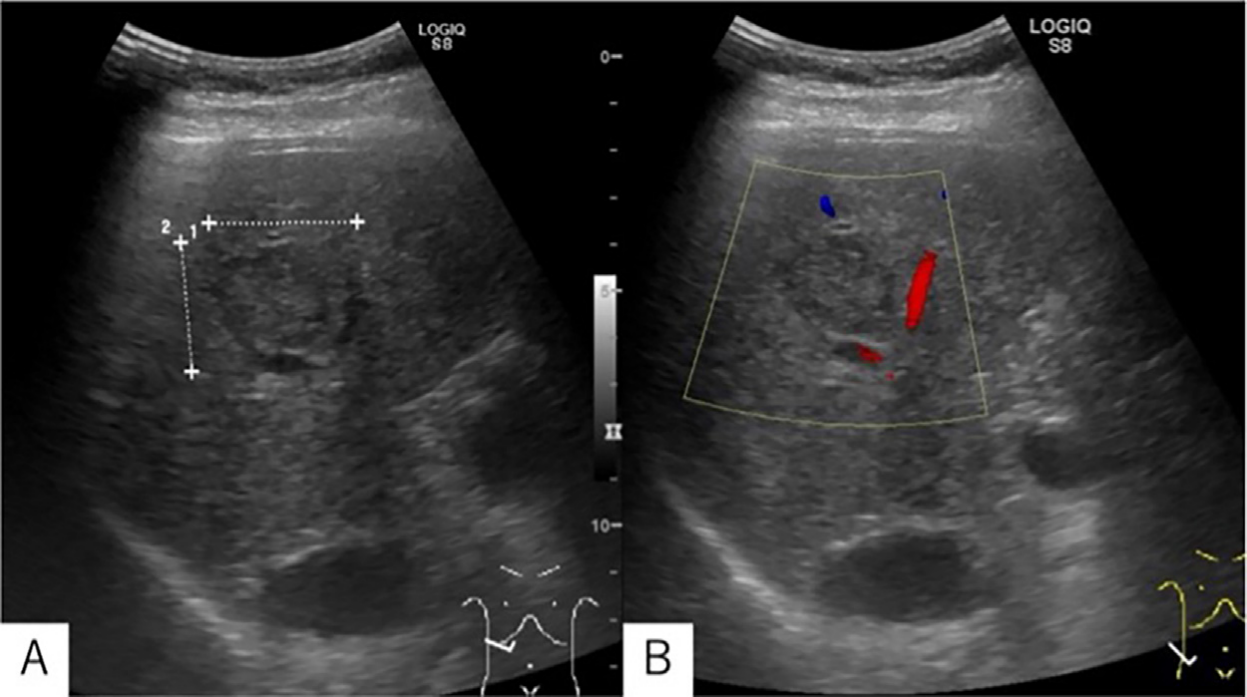
Abdominal ultrasound performed during a routine health checkup revealed a 3 cm mass-like lesion in the posterior segment of the right hepatic lobe. The lesion exhibited mixed echogenicity with both hyperechoic and hypoechoic areas but was overall isoechoic relative to the surrounding liver parenchyma. Posterior acoustic enhancement was observed. A peripheral band-like hypoechoic rim surrounded the lesion. No blood flow signals were observed in the lesion in Color Doppler, and no changes in echo intensity (chameleon sign) were observed with position changes (Not listed).

Abdominal US: A 3 cm mass lesion was detected in the posterior segment of the right hepatic lobe. The lesion appeared generally isoechoic, with a heterogeneous internal pattern consisting of high-, iso-, and low-intensity areas. Posterior acoustic enhancement was mildly increased, and the lesion was surrounded by a low-echo border. No positional change in echogenicity (chameleon sign) was observed, and no blood flow signals were detected on Color Doppler imaging (Fig. 1). As this was a newly identified lesion with features suggestive of a neoplastic process, including HCC, the patient was referred to a local hospital for further evaluation.

**Fig. 2:**
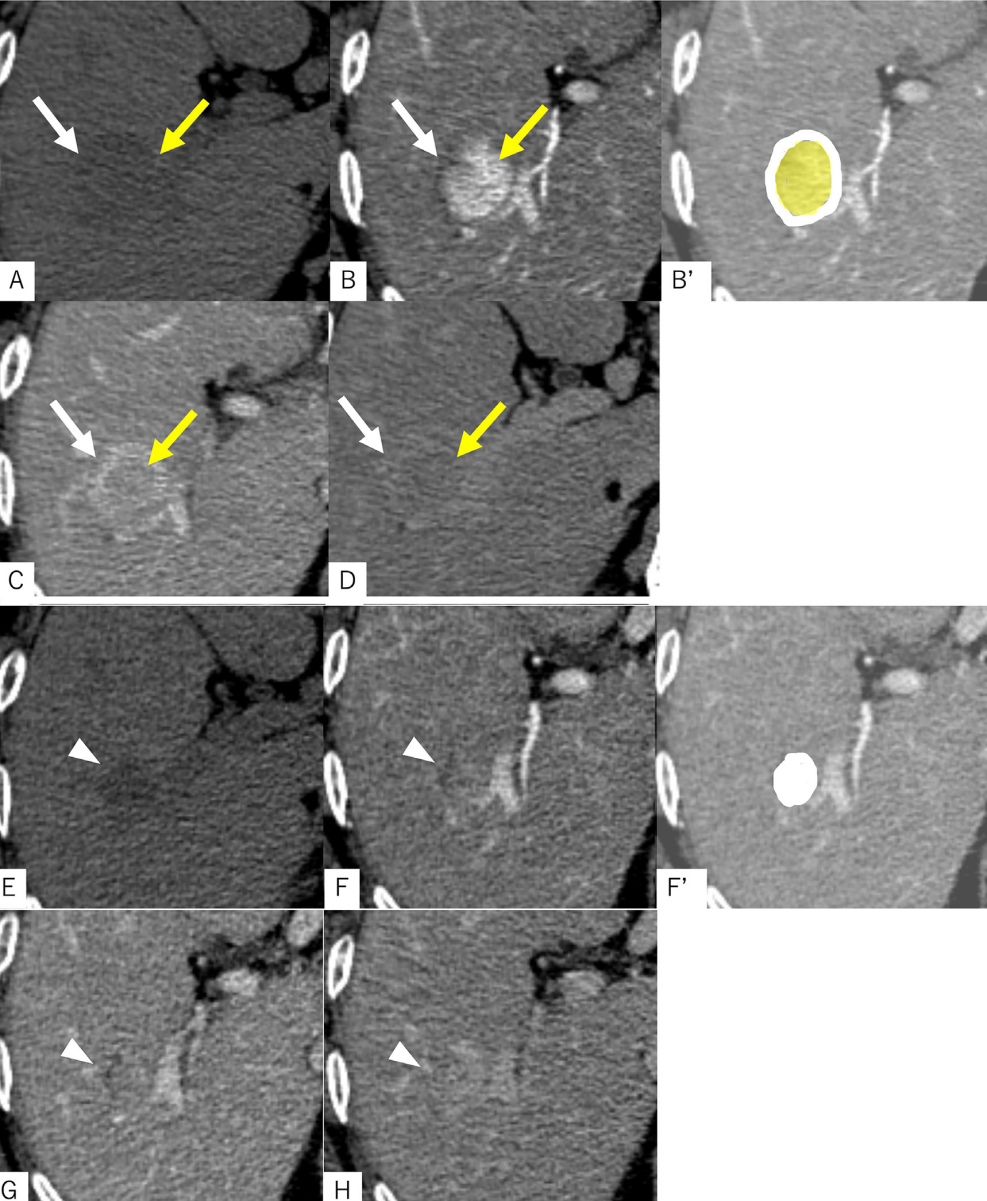
Contrast-enhanced CT images taken at the referring hospital 12 days after the initial ultrasound examination (A–D), and those obtained at our institution 40 days after the ultrasound (E–H), demonstrate notable changes in the lesion’s morphology and enhancement pattern. Initially (A–D), the lesion appeared to consist of 2 distinct components. The central area (yellow arrows) was hyperdense compared to the surrounding vasculature on non-contrast CT (A), showed intense enhancement in the late arterial phase (B), and exhibited mild washout in both the portal venous (C) and equilibrium phases (D). In contrast, the peripheral portion of the lesion (white arrows) was isodense to the adjacent vessels on non-contrast CT and remained isodense to the portal vein during the portal venous and equilibrium phases, giving it a capsule-like appearance. CT performed at our institution approximately 1 month after the initial scan (E–H) demonstrated a reduction in lesion size (arrowheads) and marked changes in internal structure. The lesion became hypodense on non-contrast CT (E), and the early arterial enhancement previously observed had diminished. Instead, a gradual and faint enhancement was noted throughout the lesion during the arterial (F), portal venous (G), and equilibrium (H) phases, suggesting a shift in vascular characteristics over time. Figures B′ and F′ are schematic illustrations corresponding to the lesions shown in images B and F, respectively. In B′, the white-shaded area represents the peripheral portion of the lesion, while the light yellow-shaded area corresponds to the central portion. In F′, the white-shaded area indicates the entire extent of the lesion.

Multiphase contrast-enhanced CT: The background liver exhibited a blunted edge and irregular surface, consistent with cirrhosis. A CT scan performed at the referring hospital 12 days after the screening ultrasound revealed a lesion with 2 distinct components. The central area appeared hyperdense on non-contrast CT, denser than adjacent blood vessels, and demonstrated hyperenhancement relative to the surrounding liver parenchyma during the late arterial phase. This enhancement persisted into the equilibrium phase. Although the lesion did not fulfill the LI-RADS definition of “washout” (as it did not become hypoattenuating compared to the liver parenchyma), the density within the lesion gradually decreased during the equilibrium phase. The peripheral component exhibited similar density to adjacent vessels on non-contrast imaging and showed enhancement comparable to the portal vein in both portal and equilibrium phases, producing a capsule-like appearance. No early venous drainage was observed. According to LI-RADS criteria, the lesion met LR-5 classification: it demonstrated nonrim arterial phase hyperenhancement (APHE), an enhancing capsule, measured over 20 mm in diameter, and was a newly identified lesion. These findings strongly suggested HCC. However, due to the absence of clear washout and the presence of persistent enhancement, a fibrotic HCC subtype, such as scirrhous-type HCC, was considered. The patient was referred to our hospital for surgical resection. However, a follow-up CT performed 40 days after the initial ultrasound revealed substantial changes. The previously observed late APHE had diminished, and a gradual, diffuse increase in contrast enhancement was noted across the lesion. Given that spontaneous necrosis can occasionally occur in HCC (3), we suspected that this lesion had undergone such necrosis (Fig. 2).

**Fig. 3:**
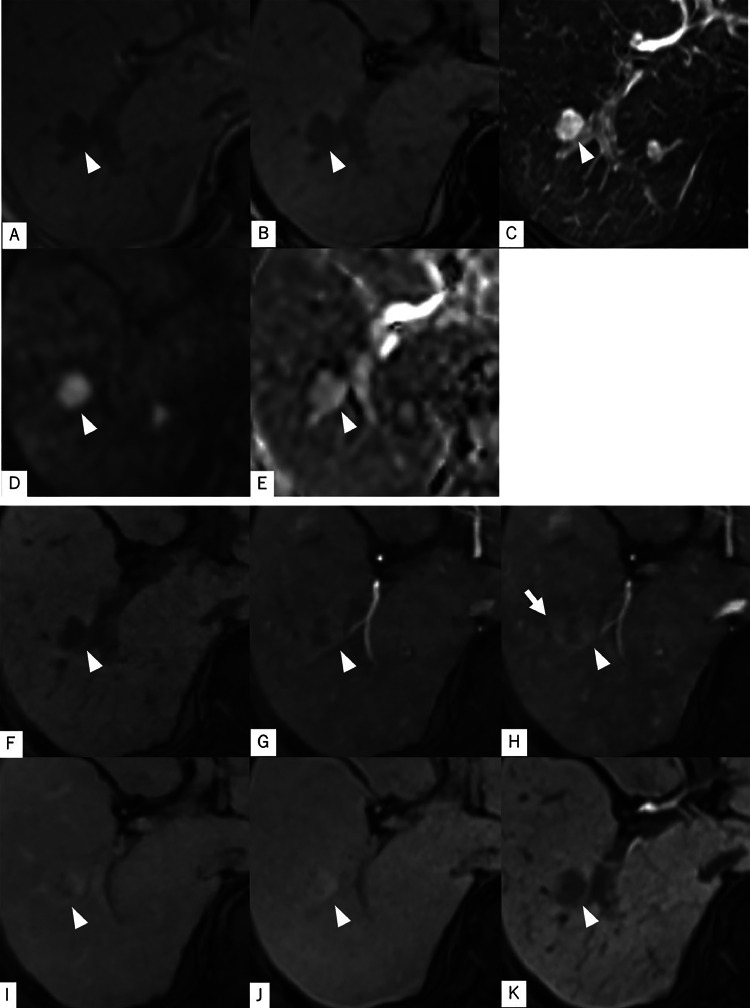
There was no signal reduction in the comparison of the in-phase (A) and opposed-phase (B) images of T1WI. In T2WI (C), a clear high signal was observed, consistent with the high signal intensity typically seen in solid mass lesions, such as HCC. In DWI (D), there was a high signal, but it also showed a high signal on the ADC map (E), which was considered T2 shine through, rather than diffusion restriction. On dynamic contrast enhancement, we observed a gradual contrast effect spreading from the left side of the lesion to the entire lesion from the early arterial phase (G) to the transition phase (J). In the hepatobiliary phase (K), it showed a lower signal than the surrounding liver. In the late arterial phase (H), a localized enhancement around the lesion, which did not show any signal change on plane MRI, was observed, raising suspicion of an AP shunt or drainage blood flow from the tumor (corona enhancement).

Gadoxetic acid-enhanced magnetic resonance imaging (MRI): A gadoxetic acid-enhanced MRI was performed at our hospital 42 days after the initial ultrasound. On T1-weighted imaging (T1WI), the lesion demonstrated lower signal intensity than the surrounding liver parenchyma. No signal drop was observed between the in-phase and opposed-phase T1WI, indicating the absence of fat deposition. T2-weighted imaging (T2WI) revealed a markedly high signal intensity. Although this was higher than typically seen in HCC, we interpreted it as reflecting degenerative changes associated with spontaneous necrosis, in correlation with the previous contrast-enhanced CT findings. Diffusion-weighted imaging (DWI) also showed high signal intensity, and the apparent diffusion coefficient (ADC) map similarly demonstrated high signal. These findings suggested that the hyperintensity on DWI was not due to true diffusion restriction but rather a T2 shine-through effect from the high T2WI signal. On contrast-enhanced MRI, we observed a gradual increase in contrast enhancement from the early arterial phase through the transitional phase, extending from the left side of the lesion to its entirety. Although there were no signal changes on non-contrast MRI, a hyperintense area was noted only in the late arterial phase, extending from the lesion margin into the adjacent liver parenchyma. This was suspected to represent an arterioportal (AP) shunt or drainage flow from the lesion (corona enhancement). In the hepatobiliary phase, the lesion remained hypointense relative to the surrounding liver tissue (Fig. 3).

Despite the reduction in tumor size over time, the imaging findings, particularly from the previous hospital, led us to suspect scirrhous-type HCC with degenerative changes due to spontaneous necrosis. Therefore, surgical resection was performed 12 days after the MRI.

**Fig. 4:**
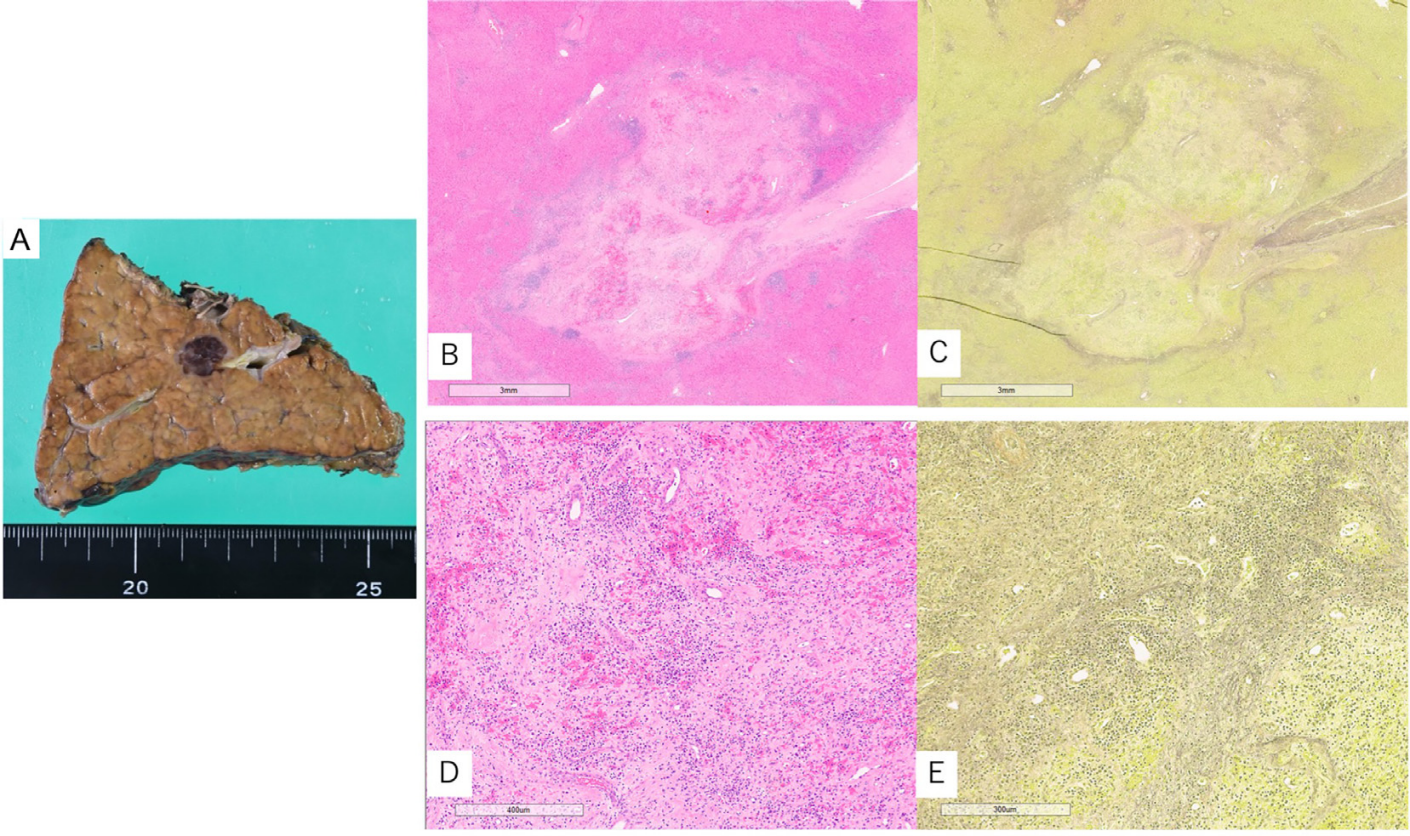
Gross observation of the resected specimen (A) revealed a dark red, organizing, thrombus-like mass. Histopathological examination with hematoxylin and eosin (H&E) staining (B) and Elastica van Gieson (EvG) staining (C) demonstrated fibrosis with inflammatory cell infiltration and organizing features. Hepatocyte staining was negative (not shown). Numerous muscularized vascular structures were identified within the lesion (D, H&E), and abnormal arterial proliferation was noted in the surrounding portal venous area (E, EvG).

Pathological examination: Gross examination of the resected specimen revealed that the center of the mass was dark red in color, resembling a coagulated blood clot. No hepatocyte-derived tissue staining positive for hepatocyte markers was identified within the lesion. Furthermore, there was no evidence of coagulative necrosis, which is typically observed in spontaneous necrosis of HCC. The lesion exhibited varying degrees of muscular vascular proliferation, and the portal venous region surrounding the lesion showed abnormal arterial development. Many of these arteries had thickened walls and narrowed lumens, suggesting ischemic changes. Additionally, there was inflammatory cell infiltration and fibrotic proliferation with sclerosis, findings characteristic of hepatocyte loss and fibrosis seen in segmental atrophy. Histological evaluation at the time of surgery confirmed segmental atrophy. Uneven distribution of portal venous structures was also noted in the peritumoral liver parenchyma (Fig. 4).

## Discussion

Segmental atrophy of the liver is a benign condition characterized by progressive parenchymal loss in a hepatic segment or subsegment, often accompanied by fibrosis, bile duct proliferation, and vascular abnormalities [4,5]. On imaging, it can appear as a mass-like lesion with capsular retraction, delayed enhancement, and volume loss—features that closely mimic hepatic malignancies such as HCC, cholangiocarcinoma (CCC), and metastatic tumors. Previous reports have highlighted these misleading imaging [6,7], which frequently lead to misdiagnosis and unnecessary surgical intervention. Pathological studies have proposed a classification system for hepatic segmental atrophy, describing 4 progressive histological stages [5]: (1) early parenchymal collapse with bile duct proliferation, (2) reduced bile ducts with increased elastosis, (3) nodular elastosis with scattered residual hepatocytes, and (4) dense fibrous nodules often containing biliary cysts and vascular changes. This progression underscores the dynamic nature of the lesion, evolving from inflammatory and regenerative changes to advanced fibrosis. The variation in histological features across these stages contributes to the diversity of imaging appearances, making accurate radiologic diagnosis particularly challenging.

In our case, the lesion initially exhibited strong arterial enhancement and delayed peripheral enhancement—features suggestive of a capsule and characteristic of scirrhous-type HCC. However, histopathological examination revealed segmental hepatic atrophy with marked vascular proliferation. These findings suggest that the early and persistent enhancement may have reflected the lesion’s original composition before the onset of atrophy. Additionally, the delayed peripheral enhancement likely corresponded to peripheral fibrotic changes, consistent with early to intermediate stages of segmental atrophy.

MRI findings included high signal intensity on T2-weighted and DWI, but without diffusion restriction on ADC maps. This pattern likely represents the final stage of the atrophic process, in which fibrotic tissue has replaced the normal hepatic parenchyma.

Given the observed reduction in lesion size over time, the lesion was initially suspected to be a spontaneously necrotic HCC. However, coagulative necrosis, typical of spontaneous HCC necrosis, generally appears as high signal intensity on T1WI and as hyperattenuation on non-contrast CT [3], neither of which was present in this case. Retrospective analysis, therefore, suggested that the lesion's imaging characteristics were not consistent with coagulative necrosis.

To our knowledge, although hepatic segmental atrophy has been previously reported, no cases have detailed the temporal evolution of imaging findings during the atrophic progression. This case may represent such a progression, incidentally captured at a stage when only the peripheral portion of the lesion was undergoing degeneration.

Notably, the patient had underlying PBC. In PBC, portal perfusion imbalance and elevated portal pressures can occur even in early stages, potentially leading to regional ischemia and atrophic changes. These hemodynamic changes are known to cause focal hypervascular lesions, such as focal nodular hyperplasia (FNH) [8,9]. In our case, pathology showed uneven blood flow in the liver tissue around the lesion, suggesting that this may have led to a hypervascular pseudolesion similar to FNH-like lesion. As FNH typically receives blood flow radially from a central artery [10], ischemic changes may preferentially affect the periphery, resulting in an outward-to-inward atrophic pattern. In this case, peripheral atrophy was visualized as a band of delayed enhancement at the margins of the early-enhancing lesion, mimicking a capsule and giving rise to an HCC-like appearance.

Preoperative imaging met the LI-RADS criteria for HCC (LR-5), including nonrim APHE, the presence of an enhancing “capsule,” lesion size ≥20 mm, and the impression of a newly appearing lesion [2]. However, the putative capsule measured approximately 5 mm in thickness relative to a 3 cm lesion, which is thicker than what is typically observed in HCC capsules [11]. Additionally, the classification of the lesion as “new” was based solely on ultrasound screening, without prior CT or MRI for objective comparison. As ultrasound is highly operator-dependent and lacks reproducibility, the possibility that the lesion was pre-existing but previously undetected cannot be excluded.

The imaging findings of segmental atrophy of the liver can closely mimic those of malignant hepatic tumors, making differentiation particularly challenging. The most critical differential diagnosis is HCC, especially when the atrophic segment exhibits APHE and delayed peripheral enhancement suggestive of a fibrous capsule. However, unlike typical HCC, segmental atrophy often lacks diffusion restriction on ADC maps and does not demonstrate true washout or intralesional fat on unenhanced imaging. Several reports have noted that segmental atrophy typically shows no significant FDG uptake on PET imaging [6], which may support its benign nature. However, it is important to recognize that HCC, particularly well-differentiated subtypes, can also demonstrate low or absent FDG accumulation. Therefore, the absence of FDG uptake is not a reliable discriminator between segmental atrophy and HCC, and FDG-PET alone cannot be considered a definitive diagnostic tool in such cases. Cholangiocarcinoma may also resemble segmental atrophy when it presents with peripheral rim-like enhancement and delayed central enhancement. However, cholangiocarcinoma is typically associated with bile duct involvement and peripheral bile duct dilatation, features not seen in segmental atrophy. Inflammatory pseudotumors may demonstrate variable enhancement and high signal intensity on T2WI, mimicking the inflammatory changes associated with atrophy. However, they are often accompanied by systemic symptoms. Segmental hepatic infarction or ischemia may also present as focal volume loss with delayed enhancement; however, these lesions are typically wedge-shaped [12] and are usually associated with acute clinical symptoms or identifiable vascular occlusion. Awareness of these entities and their distinguishing imaging features is essential to avoid misdiagnosis and prevent unnecessary surgical intervention for benign conditions such as segmental atrophy. To further clarify the imaging differences between segmental hepatic atrophy and HCC, we have summarized their distinguishing features in Table 1 [4,6]. This comparison may help improve diagnostic accuracy and reduce the risk of misdiagnosis in clinical practice.

**Table 1:** Imaging characteristics distinguishing hepatic segmental atrophy and HCC.

	Hepatic segmental atrophy	HCC
T2WI	Mild to marked hyperintensity	Mild hyperintensity
DWI/ADC	Hyper/hyperintensity (T2 shine through)	Hyper/hypointensity (diffusion restriction)
DCE-MRI	Early enhancement is seen depends on histological spectrum, peripheral rim enhancement and washout are uncommon	Early enhancement, washout and peripheral rim enhancement are common
Hepatobiliary phase	Hypointensity	Commonly hypointensity; rarely iso- or hyperintensity
Fat (based on PCI)	Absent	May be present
FDG-PET	None to liver-equivalent uptake	Variable uptake depending on differentiation

DCE-MRI, dynamic contrast-enhanced MRI; PCI, phase contrast imaging.

## Conclusion

We report a rare case of hepatic segmental atrophy that closely mimicked HCC on imaging. This case is notable for potentially capturing a dynamic progression of the atrophic process, in which early peripheral degeneration resembled the morphological features of HCC. Segmental atrophy can display diverse enhancement patterns and mass-like appearances that overlap with those of hepatic malignancies, particularly in patients with underlying liver disease. Careful assessment of atypical features—such as the absence of true washout, lack of diffusion restriction, disproportionately thick capsule-like structures, and progressive morphological changes—may assist in differentiating segmental atrophy from malignant tumors. Improved recognition and understanding of the evolving imaging characteristics of hepatic atrophy may help avoid misdiagnosis and prevent unnecessary surgical interventions in similar future cases.

## Patient consent

Written informed consent was obtained from the patient for the publication of this case report and the accompanying images.
